# Relationships Between Environmental Conditions And Fish Assemblages In Tropical Savanna Headwater Streams

**DOI:** 10.1038/s41598-020-59207-9

**Published:** 2020-02-07

**Authors:** Thiago Bernardi Vieira, Francisco Leonardo Tejerina-Garro

**Affiliations:** 1 Laboratório de Ictiologia de Altamira – LIA, Universidade Federal do Pará (UFPA), Campus Altamira. Rua Coronel José Porfírio 2515, São Sebastião, Altamira, PA CEP 68372-040 Brazil; 2 Programa de Pós-Graduação em Biodiversidade e Conservação – PPGBC, Universidade Federal do Pará (UFPA), Campus Altamira. Rua Coronel José Porfírio 2515, São Sebastião, Altamira, PA CEP 68372-040 Brazil; 3 Laboratório de Biodiversidade, Programa de Mestrado em Sociedade, Tecnologia e Meio Ambiente, UniEVANGÉLICA, Av. Universitária km. 3,5, Cidade Universitária, Anápolis, GO CEP 75083-515 Brazil; 4 Centro de Biologia Aquática, Escola de Ciências Agrárias e Biológicas, Pontifícia Universidade Católica de Goiás - Campus II, Av. Engler s/n, Jardim Mariliza, Goiânia, GO CEP 74605-010 Brazil

**Keywords:** Ecology, Community ecology, Limnology

## Abstract

Riparian vegetation plays an important role in providing energy to small watercourses and maintaining ecological processes through organic matter input and together with hydrological and geomorphological watercourse characteristics influence on fish assemblages. The goal of this paper was partitioning and quantifying the influence of riparian zone (type of riverbank substrate, bank slope, type of riparian vegetation cover and percentage of riparian vegetation cover on the main channel), physical habitat (stream channel width and depth, type of substrate and aquatic habitat in channel, water velocity and organic matter), water quality (turbidity, temperature, conductivity, pH, dissolved oxygen and chlorophyll concentration) and spatial variables (linear distances between sampled points) on fish assemblages (richness and abundance per species) in headwater streams of the Upper Paraná River basin, Central Brazil. For this purpose, it was performed a variation partitioning analysis between riparian, physical habitat, water and spatial variables sets and a Redundancy Analysis to quantify the influence of variables on the fish assemblages. Only the physical habitat and water quality variables influenced the fish assemblages (richness and abundance per species).

## Introduction

Freshwater fish assemblages are structured by variables related to both water quality and riparian vegetation^1–6^. In this sense, warmer waters exhibit higher fish abundance and biomass while highly oxygenated waters may lead to greater species diversity^7–9^. Riparian vegetation is a transitional semiterrestrial system^10^ that provides energy in watercourses through the input of organic matter^2^. Leaves deposited on the watercourse bed contribute indirectly to fish food because they act as a substrate for numerous microorganisms^11^ and insects^12,13^. In addition, riparian trees and roots restrict channel widening, cause channel deepening and add coarse woody debris favoring fish concealment and channel complexity.

The influences of water and riparian vegetation on fish assemblages are not independent^2,10,14,15^; that is, riparian vegetation may directly or indirectly influence water variables^16^. For example, water temperature is directly influenced by riparian vegetation, which regulates the watercourse insolation level^17,18^ and influences primary production^19^. Conversely, channel depth and substrate heterogeneity are indirectly influenced by riparian vegetation because the riparian zone regulates the entry of sediment that can be deposited into the watercourse^10,20,21^.

Another factor that should not be neglected is the spatial factor (e.g., the river network), which includes geographical barriers that hamper or prevent species migration between locations. Abundance and richness are diversity metrics that are spatially structured^22–28^. Spatial factors are a consequence of the geological and local climatic influence on the streams in a river network^29–31^ and the position of the watercourse along a longitudinal gradient (upstream-downstream^32^) for the 1^st^-3^rd^ ^33^ and 4^th^-7^th^ order^34^ streams. A spatial model coupled with a river network accurately explains fish richness patterns^35,36^. Additionally, the river network acts as a corridor^37^^,^ facilitating fish species dispersion^38^ or acting as a filter^39^.

Furthermore, the individual influence of water, riparian or spatial processes on the structure of fish assemblages is not necessarily consistent^40^. Instead, the influence of these processes results more often from their interaction^41^. Therefore, physical habitat variables influence fish assemblages either alone^27^ or in combination with water quality variables^42^.

The aim of this paper was to partition and quantify the influence of riparian, physical habitat, water quality and spatial variables on fish assemblages in headwater streams located in the Upper Paraná River basin, Central Brazil.

## Results

A total of 4879 specimens belonging to 59 species and 19 families were collected (Table 1).

**Table 1:** Number of individuals (n) and fish species collected in the stream sites sampled in the Upper Paraná River basin, Central Brazil, between April and September 2009.

ORDER	n	ORDER	n
Family		Family	
*Specie*		*Specie*	
CHARACIFORMES		PERCIFORMES	
Anostomidae		Cichlidae	
*Leporinus microphtalmus* Garavello, 1989	57	*Cichla kelberi* Kullander & Ferreira 2006	2
Characidae		*Cichlasoma paranaense* (Kullander, 1983)	19
*Astyanax altiparanae* Garutti & Britski, 2000	615	*Crenicichla niederleinii* (Holmberg, 1891)	30
*Astyanax eigenmanniorum* (Cope, 1894)	240	*Oreochromis niloticus* (Linnaeus, 1758)	2
*Astyanax fasciatus* (Cuvier, 1819)	679	*Coptodon rendalli* (Boulenger, 1897)	11
*Astyanax scabripinnis* (Eigenmann, 1927)	356	SILURIFORMES	
*Astyanax* sp. 1	1	Aspredinidae	4
*Astyanax* sp. 2	1	*Bunocephalus coracoideus* Cope, 1874	4
*Bryconamericus stramineus* Eigenmann, 1908	728	Auchenipteridae	2
*Knodus* sp.	19	*Tatia neivar* (Ihering, 1930)	2
*Oligosarcus planaltinae* Menezes & Géry, 1983	16	Callichthyidae	
*Piabina argentea* (Reinhardt,1867)	401	*Aspidoras fuscoguttatus* Nijssen & Isbrücker 1976	369
*Planaltina myersi* Böhlke, 1954	18	*Corydoras flaveolus* Ihering, 1911	17
*Serrapinnus* sp.	27	Heptapteridae	
Crenuchidae		*Cetopsorhamdia iheringi* Schubart & Gomes, 1959	24
*Characidium fasciatus* (Britski, 1970)	31	*Cetopsorhamdia* sp.	33
*Characidium gomesi* (Travassos, 1956)	36	*Heptapterus mustelinus* (Valenciennes, 1835)	1
*Characidium* sp.	14	*Imparfinis longicauda* Borodin 1927	5
*Characidium zebra* (Eigenmann, 1909)	51	*Imparfinis schubarti* (Gomes, 1956)	21
Curimatidae		*Imparfinis* sp.	3
*Cyphocharax modestus* (Fernández-Yépez, 1948)	2	*Phenacorhamdia* sp.	4
*Steindachnerina insculpta* (Fernández-Yépez, 1948)	200	*Phenacorhamdia tenebrosa* (Schubart 1964)	
Erythrinidae		*Pimelodella* sp.	49
*Hoplias malabaricus* (Bloch, 1794)	9	*Rhamdia quelen* (Quoy & Gaimard, 1824)	147
Lebiasinidae		Loricariidae	
*Pyrrhulina australis* Eigenmann & Kennedy, 1903	1	*Hisonotus* sp.	2
Parodontidae		*Hypostomus ancistroides* (Ihering, 1911)	168
*Apareiodon ibitiensis* (Amaral Campos, 1944)	70	*Hypostomus* cf. *strigaticeps*	2
*Apareiodon vladii* (Pavanelli, 2006)	1	*Hypostomus plecostomus* (Linnaeus, 1758)	5
*Parodon nasus* Kner, 1859	35	*Hypostomus regani* (Ihering, 1905)	44
Prochilodontidae		*Hypostomus* sp. 1	28
*Prochilodus lineatus* (Valenciennes, 1836)	3	*Hypostomus* sp. 2	16
Poeciliidae		*Hypostomus* sp. 3	46
*Poecilia reticulata* Peters, 1859	133	*Loricaria* sp.	2
GYMNOTIFORMES		*Rineloricaria latirostris* (Boulenger, 1900)	13
Gymnotidae		Trichomycteridae	1
*Gymnotus carapo* Linnaeus, 1758	23	*Trichomycterus* sp.	1
Sternopygidae		SYNBRANCHIFORMES	8
*Eigenmannia trilineata* López & Castello, 1966	11	Synbranchidae	8
		*Synbranchus marmoratus* Bloch, 1795	8
Total			4879

### Influence of the environmental conditions on fish assemblages

**Figure 1:**
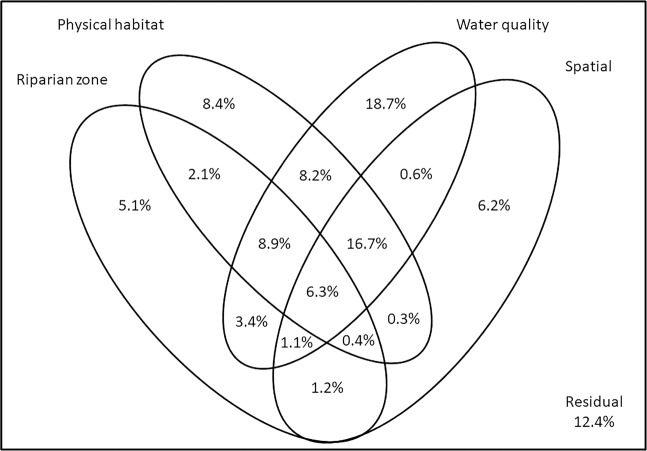
Variation partitioning (percentage) of stream fish richness among physical habitat, water quality, riparian zone and spatial compartments.

**Figure 2:**
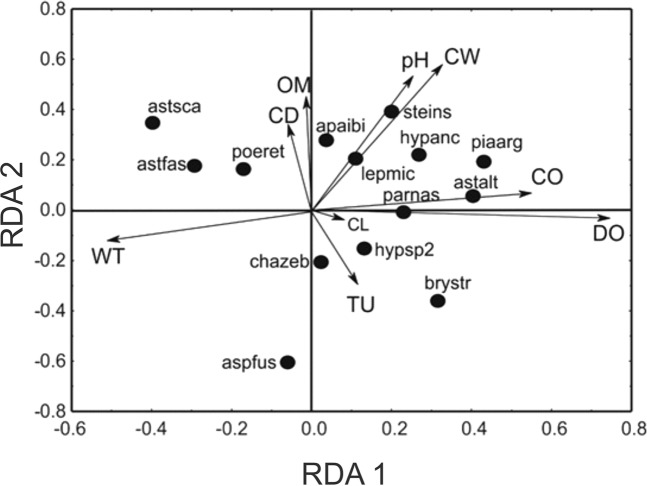
Analyses of redundancy (RDA) output correlating stream fish assemblage to environmental water variables. aspfus = *Aspidoras fuscoguttatus*; astalt = *Astyanax altiparanae*; astfas = *Astyanax fasciatus*; astsca = *Astyanax scabripinnis*; brystr = *Bryconamericus stramineus*; poeret = *Poecilia reticulate*; piaarg = *Piabina argentea*; steins = *Steindachnerina insculpta*; CO = conductivity; CL = chlorophyll concentration; MO = organic matter; CW = channel width; TU = turbidity; DO = dissolved oxygen; CD = channel depth; WT = water temperature. P1 – P22 = stream sites. Only species with >90.0% of contribution to the structure of RDA are represented.

The variation partitioning analysis indicated that fish abundance variation is explained by water quality (18.7% of variation), physical habitat (8.4%), spatial (6.2%) and riparian zone variables (5.1%; Fig. 1). The interactions among the spatial variables, water quality and physical habitat explain 16.7% of the variation, those among the physical habitat, riparian zone and water quality explain 8.9%, those between the physical habitat and water quality explain 8.2%, and the other interactions represent ≤3.4% (Fig. 2). The Procrustes analyses indicated a significant correlation between the fish abundance and the physical habitat (M^2^ = 0.295; p < 0.001) and water quality variables (M^2^ = 0.565; p < 0.001) and no significance for the riparian zone (M^2^ = 0.200; p = 0.526) or spatial variables (M^2^ = 0.150; p = 0.744). All the non-metric multidimensional scaling NDMS analyses performed had a good fit (stress < 0.02).

### Fish assemblages-environmental conditions relationships

According to the broken stick criteria, there were two significant axes for the PCAs performed separately on the water quality (77% of the variation) and physical habitat (81% of the variation), eight axes for the PCA performed on the riparian zone (87% of the variation) and three significant MENs (Moran’s Index = 0.01 for each one) for the spatial variables.

The multiple linear regression showed no significant relation between fish abundance and the variables of the four groups considered (r^2^ = 0.566; F _(12, 27)_ = 2.536; p = 0.128). In contrast, a significant relation was observed between fish richness (r² = 0.784; F _(12, 27)_ = 5.865; p = 0.001) and the PCA-1 of the physical habitat variables (p = 0.005; Table 2). All of the other compartments did not display significant relationship (Table 2).

**Table 2:** Multiple regression statistics between the fish richness attribute and the variables of the physical habitat (PH), water quality (W), riparian zone (RZ) and spatial (SP) and compartments represented by principal component axes (PCA); see the methodological section for more details. The contribution of each variable is displayed. SC = Standard coefficient; VIF = Variable Inflation Factor; *t = *Student *t* test. *Significant probabilities (p < 0.05).

Fish attribute	Variable	Coefficient	SC	VIF	*t*	p
Richness	Intercept	16.607	—	1.214	13.68	0.001*
	PCA W1	−0.352	−0.065	1.511	−0.233	0.819
	PCA W2	−1.731	−0.035	10.51	−0.165	0.872
	PCA PH1	3.821	0.609	1.32	2.896	0.005*
	PCA PH2	−23.663	−0.473	9.262	−2.555	0.064
	PCA RZ1	0.854	0.269	0.717	1.192	0.255
	PCA RZ2	−1.547	−0.400	0.632	−2.449	0.092
	PCA RZ3	−0.889	−0.183	0.734	−1.211	0.247
	PCA RZ4	−1.171	−0.238	0.746	−1.57	0.140
	PCA RZ5	−0.777	−0.139	1.043	−0.745	0.469
	PCA RZ6	1.553	0.257	0.906	1.714	0.110
	PCA RZ7	−1.455	−0.217	0.963	−1.51	0.155
	PCA RZ8	−2.351	−0.344	1.186	−1.982	0.069

The relationship between assemblage and physical habitat variables is detailed by the RDA (total variance explained by the two axes = 53.4%; F _(10, 17)_ = 3.543; p = 0.003). The first axis (35.17%) was positively correlated with conductivity, dissolved oxygen and chlorophyll concentration and negatively correlated with water temperature, whereas the second axis (18.23%) was positively correlated with organic matter, channel depth, pH and channel width and negatively correlated with turbidity (Fig. 2). The characins *Piabina argentea* and *Astyanax altiparanae* and the scrapetooths *Parodon nasus* were related to high values of water conductivity, dissolved oxygen and chlorophyll concentration, whereas the characins *Astyanax fasciatus* and *Astyanax scabripinnis* and the poeciliid *Poecilia reticulata* were associated with elevated water temperature values. The scrapetooths *Apareiodon ibitiensis*, the headstander *Leporinus microphthalmus*, and the toothless *Steindachnerina insculpta* were associated with elevated organic matter and pH values and a large and deep channel stream. The characin *Bryconamericus stramineus*, the callichthyid armored catfish *Aspidoras fuscoguttatus* and the South American darter *Characidium zebra* were correlated with high values of turbidity (Fig. 2).

## Discussion

The riparian zone does not display any significant influences on fish abundance or richness in the headwater streams sampled. Similar results using a different methodology were obtained for fish diversity^43^ in 1^st^ to 3^rd^ order headwaters streams in the Amazon region. This result suggests a low influence of riparian vegetation removal, assessed indirectly in this paper by the variables of the riparian zone group (type and percentage of the vegetation cover), on fish assemblages. However, studies focused on this subject have stressed the influence of the riparian zone on fish assemblages in the Amazon (channel fragmentation, deforestation^44^; mechanized agriculture^43^), São Francisco (deforestation^42,45^) and Paraná River basin (deforestation^45^), the last two of which contain the same vegetation cover of the area sampled in this paper (i.e., Cerrado).

The spatial component also showed no significant influence on fish assemblages. The abundance and richness of plants and animals, including stream organisms, are spatially structured^45,46^ because of the influence of geology, the local climate^30^ and the watercourse position along a longitudinal gradient^32^, especially for 1^st^ to 3^rd^ order streams^33^. However, if the 1^st^ and 2^nd^ order streams sampled in this study were in the same geologic (a combination of Precambrian metamorphic rocks, continental sedimentary rocks and tholeiitic basalts^47^) and climatic (tropical climate with a dry season) domain, a similarity of fish abundance and richness could be expected. It suggest that the influence of environmental conditions and resources appear to be more influent than the spatial process, even that the sample sites are located in different basins.

In this study, fish richness was influenced by physical habitat (stream channel width and depth, and organic matter) and water quality (conductivity, water temperature, pH, chlorophyll, dissolved oxygen, and turbidity) variables. These variables are known to structure not only fish assemblages^4,48,49^ but also their specific attributes, such as richness^50–53^. The results agree with those reported for Amazonian^43^ and Cerrado fish assemblages of 1^st^ to 3^rd^ order headwater streams^42^, although some previous studies did not separate the influence of physical habitat and water quality variables from those of the riparian zone, as was done in this paper. Additionally, these physical habitat and water quality variables are better predictors of fish assemblage variability than riparian or catchment variables^43^ or land use and the geophysical landscape^42^ in Amazon and Cerrado headwater streams, respectively.

The influence of water conductivity on fish assemblages, as observed in this study, was also reported for tropical^54^ and temperate watercourses^51^. Conductivity is a surrogate or correlate of water productivity, which influences freshwater fish body condition^45^, because it measures the electrical conductivity resulting from the concentration of dissociated ions^55^. Fish species can prefer aquatic habitats with specific requirements, such as elevated values of water conductivity, dissolved oxygen and chlorophyll concentration (as seen in the scrapetooths *Parodon nasus* and the characins *Astyanax altiparanae* and *Piabina argentea* in the watercourses sampled). In the case of *P. nasus*, the relationship observed is explained because this species is found in riffles^56^ where there are elevated levels of dissolved oxygen. Furthermore, *P. nasus*, a periphyton scraper that prefers rocky substrates where algae and bryophytes are abundant, is associated with waters with high conductivity because of eutrophication^57^. On the other hand, the characin *A. altiparanae* is considered tolerant to aquatic environmental changes and disturbances such as pollution^58^, which elevates water conductivity, and displays adaptations (i.e., a projection of the lower lip increase oxygen capture from water surface) to survive in low concentrations of dissolved oxygen^56^. Finally, the characin *P. argentea* is a midwater swimmer described as an opportunistic generalist species abundant in disturbed watercourses (modified from lotic to lentic conditions)^59^ that is also positively correlated to dissolved oxygen concentrations in streams of the Upper Paraná River basin^60^.

The poecilid *P. reticulata*, an exotic species in Brazilian watercourses, and the characin *A. fasciatus* are tolerant to habitat alterations^57,61^. Additionally, *A. fasciatus* and *A. scabripinis* (to a lesser extent^62^) are sensitive to water temperature because of the influence on their reproduction cycles^63^, whereas *P. reticulata* displays female-choice sexual selection^64^, fry production^65^, schooling behavior^66^, and aquatic surface respiration (ASR) to meet oxygen demand in hypoxic water^67^ regulated by the water temperature. These relationships explain the affinity of these species for the water temperatures found in the streams sampled. However, this affinity, especially for *P. reticulata* and *A. scabripinis*, can change during the low- and high-water seasons, when both species are associated with low water temperature^68^.

The accumulation of organic matter, such as trunks and bundles of leaves, may be responsible for species coexistence in different habitats. This coexistence can occur because of the increase in habitat heterogeneity resulting from organic matter input^69,70^ from the surrounding riparian zone or the transport of leaves and other matter from upstream to downstream^71–75^, which are then deposited in stream areas with low water velocity^76^. This seems to be the case in this study for the scrapetooths *Apareiodon ibitiensis*, a detritivorous species that scrape the algal film adhered on the surfaces of rocks and logs^77^, the toothless characin *Steindachnerina insculpta*, a bottom feeding fish^55^, and the headstander *Leporinus microphthalmus*, which, like other anostomids, feeds on sponges, detritus, insects, seeds, leaves, and filamentous algae, in the substrate^78,79^.

Additionally, the preference of these species for relatively large and deep streams can be related to their body length (*A. ibitiensis* = 11.3 cm, *S. insculpta* = 16.1 cm, *L. microphthalmus* = 11,8 cm^54^), as reported for *A. ibitiensis*^80^. However, the results found can be influenced by local or regional modifications. For example, the fragmentation of a channel or watercourse and local/regional deforestation influence the organic matter inputs (leaves, trunks and stems in this case), habitat complexity and riverbed stability. This, in turn, influences fish richness, as pointed out for Amazonian headwater streams^44^.

Among the species sampled, the callichthyid armored catfishes *Aspidoras fuscoguttatus*, the characin *Bryconamericus stramineus* and the South American darter *Characidium zebra* are associated with high water turbidity. The callichthyid *A. fuscoguttatus* is a bottom dwelling species that swims near the watercourse substrate gathering food (“grubber excavating while moving”^81^). This behavior can explain its ability to exploit the watercourse substrates, which are covered by fine sediments^56^ that are transported by water, and its capacity to survive in streams that have remarkable seasonal oscillation in turbidity, with lower values during the dry period and higher values in the rainy period^82^. On the other hand, the characin *B. stramineus* is a predominantly insectivorous^83^ active swimmer^84^ that is abundant in shallow streams of the Upper Paraná basin with elevated turbidity^83,85^ and water velocity^85^. The relationship of *C. zebra* with water turbidity is unexpected considering that it is an indicator species of pristine environments, with a sit-and-wait behavior for capturing prey^86^ and rheophilic preferences that can be affected by high levels of suspended sediments in the water column and the resulting siltation of the substrate^54^.

Among the four groups of environmental variables considered, only those related to the physical habitat and water quality significantly influenced the richness of the fish assemblages. This influence is explained by the interaction of the fish assemblages with nine variables (conductivity, water temperature, pH, chlorophyll, organic matter, dissolved oxygen, turbidity, channel width and channel depth). These results indicate that local instream characteristics of headwater streams have more influence on fish assemblages than factors associated with the riparian zone in Cerrado river basin draining areas. The comparison between these findings and those from the Amazon River basin suggests that this influence exists regardless of the river basin and its vegetation cover (Cerrado and Amazon in this case).

## Materials and Methods

### Study area

**Figure 3:**
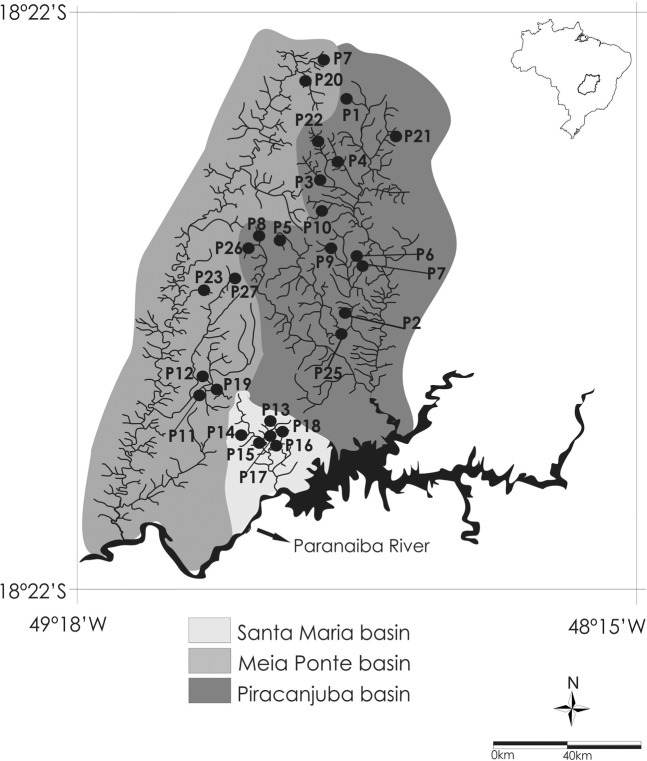
Location of streams sampled (black circles) from April to September 2009 in the Upper Paraná River basin, Central Brazil. The black area in the Paranaíba River represents the Itumbiara hydroelectric reservoir.

Twenty-seven sites (one sample site per stream) of the 1^st^ and 2^nd^ order tributaries of the Meia Ponte River (seven streams; 2.7 to 10.2 km apart from each other), Piracanjuba River (14; 4.8 to 17.8 km) and Santa Maria River (six; 4.8 to 6.0 km) were sampled, all of which are located in the Southeast Region, Goiás state, Upper Paraná River basin, Central Brazil (Fig. 3, Table 3). Sampling was conducted between April and September 2009, which corresponded with the dry season of the regional climate (Aw per the Köppen-Geiger classification). The Paraná River basin drainage is located on sedimentary deposits corresponding with the Paleozoic and Cenozoic and covered by basalt from the Jurassic-Cretaceous age^47^. The sampling stations are located on a combination of three types of rocks: i) Precambrian metamorphic rocks; (ii) continental sedimentary rocks; and (iii) tholeiitic basalts, which are abundant in the Paraná basin^47^. The vegetation cover of the Meia Ponte and Piracanjuba River basin was deciduous forest, and that of the Santa Maria basin was a semideciduous forest, all of which belong to the Cerrado (the Brazilian savanna biome).

**Table 3:** Geographic coordinates and local geomorphological characteristic of stream sites sampled between April and September 2009 in the Upper Paraná River basin, Central Brazil. MP = Meia Ponte, PI = Piracanjuba, SM = Santa Maria, SD = Standard deviation.

Basin	Stream	Coordinates		Channel				Predominant substrate
		S	W	Width (m)		Depth (m)		
				Mean	SD	Mean	SD	
MP	P7	17°25′48.0″	48°57′48.0″	0.60	0.14	0.12	0.13	Sand/Gravel/Rock
	P11	18°05′33.0″	49°21′44.0″	4.15	1.44	0.19	0.18	Rock
	P12	18°05′09.0″	49°20′44.0″	5.48	1.58	0.53	0.21	Sand
	P19	18°02′47.0″	49°21′27.0″	1.21	0.38	0.40	0.04	Gravel
	P20	17°08′19.0″	48°59′47.0″	0.98	0.23	0.10	0.08	Sand
	P23	17°21′13.0″	48°47′46.0″	4.20	1.13	0.38	0.07	Sand/Gravel
	P27	17°14′43.0″	48°55′43.0″	1.14	1.10	0.18	0.13	Sand
PI	P1	17°12′04.0″	49°03′36.0″	2.22	0.59	0.24	0.11	Sand
	P2	17°55′42.1″	48°57′28.8″	0.98	0.17	0.10	0.08	Sand/Rock
	P3	17°42′20.2″	48°54′41.9″	1.74	0.11	0.20	0.05	Sand
	P4	17°44′11.4″	48°53′35.2″	3.21	0.09	0.35	0.17	Gravel
	P5	17°40′44.0″	49°12′58.0″	4.41	0.16	0.34	0.18	Sand
	P6	17°48′21.9″	49°20′53.7″	0.69	0.83	0.15	0.18	Sand
	P8	17°45′49.6″	49°15′37.2″	1.23	0.69	0.21	0.07	Sand
	P9	17°39′58.5″	49°11′29.0″	2.16	0.68	0.32	0.19	Sand
	P10	17°39′18.4″	49°08′22.3″	4.13	1.48	0.26	0.40	Sand/Gravel
	P21	17°26′16.0″	48°56′43.0″	1.36	1.73	0.12	0.23	Sand
	P22	17°20′42.0″	48°05′08.0″	2.94	1.37	0.12	0.11	Rock
	P24	17°16′16.0″	48°02′46.0″	3.44	0.40	0.21	0.19	Sand
	P25	17°52′01.0″	48°56′31.0″	3.59	0.12	0.18	0.15	Sand
	P26	17°35′48.0″	48°56′25.0″	0.97	0.86	0.26	0.20	Sand
SM	P13	18°12′07.0″	49°09′02.0″	4.78	1.51	0.26	0.16	Gravel
	P14	18°13′03.0″	49°09′53.0″	7.78	1.40	0.51	0.18	Sand/Gravel/Rock
	P15	18°14′32.0″	49°11′27.0″	5.38	1.21	0.31	0.21	Sand/Gravel
	P16	18°12′18.0″	49°08′11.0″	4.85	1.90	0.30	0.17	Sand/Gravel
	P17	18°13′24.0″	49°14′40.0″	6.20	1.27	0.42	0.22	Sand/Gravel
	P18	18°11′45.0″	49°08′53.0″	5.92	1.34	0.35	0.07	Sand/Gravel

In each stream, one 100-m site was selected according to its accessibility, marked and georeferenced (Garmin GPSMAP64. Each site was divided into 11 transects, one every ten meters, where the data collection for both fish assemblages and variables was performed.

All sites were away from urban areas and were found in a landscape matrix formed mainly by pasture. The exception was site P17, which was surrounded by a sugarcane crop. The sites sampled had riparian vegetation covering the stream channel and at least one opening, which was intended for watering livestock or replaced by grass for feeding cattle (site P5), in the riparian cover along the site. The channel depth of the stream sites ranged from a minimum of 0.10 (P2 and P20) to a maximum of 0.53 m (P12), whereas the channel width ranged from 0.60 (P7) to 7.78 m (P14; Table 3). The predominant substrate in the sites sampled was sand, except in P4, P13, P19 (gravel) and P11 (rocky outcrops; Table 3). The predominant aquatic habitat type was lotic except in stretch P9. Upstream site P17 was located in a reservoir.

### Sampling protocols

Sixteen environmental variables were measured in each site. Six variables were associated with physical habitat, six with water quality and four with the riparian zone (Table 4).

**Table 4:** Environmental variables by compartment measured in the stream sites sampled in the Upper Paraná River basin, Central Brazil, between April and September 2009.

Compartment	Variable	Category
Physical habitat	Aquatic habitat	Pool
		Stream current
		Stream rapids
	Channel depth (cm)	—
	Channel width (m)	—
	Organic matter	Aquatic plants
		Aquatic vegetation
		Leaf pack
		Trunks and steams
		Trunks, stems and vegetation
	Stream channel substrate	Sand
		Gravel
		Mud
		Rock
	Water velocity (cm.s^−1^)	—
Water quality	Chlorophyll concentration (μg.l^−1^)	—
	Conductivity (μS.cm^−1^)	—
	Dissolved oxygen (mg.L^−1^)	—
	pH	—
	Turbidity (NTU)	—
	Water temperature (°C)	—
Riparian zone	Riverbank substrate	Clay
		Silt
		Gravel
		Mud
		Rock
	Riverbank slope	Less inclined
		Inclined
		Very inclined
	Type of riparian vegetation cover	Grass
		No coverage
		Shrubs
		Shrubs and trees
		Trees
	Percentage of riparian vegetation cover	No coverage
		Partial
		Total

Riverbank substrate, riverbank slope, aquatic habitat, type of riparian vegetation cover and percentage of riparian vegetation cover were visually characterized at each transect (along both riverbanks) along with luminosity (photometer; Polaris), stream channel width (measuring tape), stream channel depth (graduated rope) and water velocity (flowmeter; General Oceanic 2030). At the initial, middle and final transects of each site, organic matter samples of the stream channel bed and water were collected to determine algae biomass and to measure the physical and chemical variables.

Organic matter was collected using a Surber sampler (30 × 30 cm). In the laboratory, the samples were dried at 100 °C for 24 hours and weighed (SC2020 – Ohaus; 0.001 g)^87^.

Alpha chlorophyll concentration was used as a reliable and common proxy for the total phytoplankton biomass^88^, which may vary according to the degree of shading caused by riparian forests in headwater streams^19^. In the field, 25 L of water was filtered directly from the stream using a plankton net (mesh 1 μm) and a water pump (P835; Stihl). The product of the filtering process was placed in a 600 ml opaque bottle containing 1 ml of saturated magnesium carbonate. In the laboratory, the samples were filtered (cellulose ester membrane; porosity 0.45 μm) and quantified by spectrophotometry (spectrophotometer; Varian-Cary-50 CONC)^89^. The *a*, *b* and *c* chlorophyll concentrations were calculated following the Jeffrey and Humphrey equation^90^.

Water turbidity (turbidimeter; LaMotte 2020), temperature and conductivity (thermometer/conductivity meter WTW 3015i) and dissolved oxygen (DO-Lutron 5510) were measured at ~20 cm depth. The water turbidity, temperature, conductivity, dissolved oxygen and water velocity were measured at ~20 cm depth, whereas luminosity and air temperature were measured at ~20 cm above the water surface.

Fish were collected by shore electrofishing (electrofisher DC, 100–600 V plugged into a 220 V electric generator) modified from^91^; that is, the site’s length was 100 m and traversed only one time instead of being 50–80 m in length and traversed three times. Both modifications were performed based on the results of^92^, taking into account the logistics of the electrofishing gear used and displacement difficulties that occur along Cerrado streams because of physical conditions (e.g., trunks and steep stream bank). Four people collected samples for one hour in each site. The collected fish were placed in plastic bags, euthanized with a saturated clove oil solution and fixed in formalin (10%). All the bags were identified with tags containing the stream and site code. Fish was collected in the dry season when captures are more efficient because of lower water levels^93^. Fish sampling, transport and preservation of the sampled specimens were carried out in accordance with the relevant guidelines and regulations of the Sistema de Autorização e Informação em Biodiversidade, Instituto Chico Mendes de Conservação da Biodiversidade, Ministerio do Meio Ambiente (license # 20226 granted to the second author).

### Data analysis

The dataset was organized into five matrices. The first matrix was composed of species abundance (the total number of individuals per species). The second consisted of physical habitat variables (frequency values by category or average values): stream channel width and depth, stream channel substrate, aquatic habitat, water velocity and organic matter. The third consisted of water quality variables (average values): turbidity, water temperature, conductivity, pH, dissolved oxygen and chlorophyll concentration. The fourth consisted of variables related to the riparian zone (frequency values by category): riverbank substrate, riverbank slope, type of riparian vegetation cover and percentage of riparian vegetation cover in the channel. The fifth data matrix grouped the main spatial eigenvectors (MENs)^94^, which constitute a representation of the spatial process resulting from the analyses performed on the spatial data matrix (geographic coordinates) considering a linear distance (Euclidean distance) between sampling points. The MENs represent spatial autocorrelations (Moran’s index) and can be used as a surrogate for the dispersion ability of species^94,95^. Significant MENs were considered those with Moran’s index values < 0.05. All the procedures to obtain the MENs were performed in SAM macroecology software^96^.

To determine the influence of the variable groups (physical habitat, water quality, riparian zone and spatial) (environmental variables) on the fish (biotic structure), a variation partitioning analysis was performed. After that, each data matrix was transformed to a similarity matrix using a specific index (Bray-Curtis for fish species abundance and Euclidean distance for all the other data matrices) and nonparametric multidimensional scaling (NMDS) was performed^97^. Using the resulting NMDS, a correlation (Procrustes analysis^98^) was performed separately between the fish assemblages and the physical habitat, water quality, riparian zone and spatial groups (9999 permutations^99^).

To determine the relationship between the fish assemblages and the variable groups (physical habitat, water quality and riparian zone), two multiple linear regressions were performed: the first one was for fish species richness, and the second one was for fish species abundance. A principal component analyses (PCA) was performed separately on each variable’s group (physical habitat, water quality, riparian zone). The significant axes were retained based on the broken stick criteria and used to perform the multiple linear regressions. The PCA axes were used in place of the original variables to avoid multicollinearity.

Finally, redundancy analyses (RDA), which consider the percentage of explained variation (*R²*) followed by a bootstrap procedure^100^, were performed to test the interaction between fish and the physical habitat, water quality and riparian zone groups. These analyses were performed only for the data matrices with significant relationships with the fish matrices (abundance and/or richness). All the statistical analyses were performed in R software using the *vegan* package^98^.
